# Arabidopsis GAAP1 and GAAP3 Modulate the Unfolded Protein Response and the Onset of Cell Death in Response to ER Stress

**DOI:** 10.3389/fpls.2018.00348

**Published:** 2018-03-16

**Authors:** Kun Guo, Wei Wang, Weiwei Fan, Zhiying Wang, Manli Zhu, Xiaohan Tang, Wenting Wu, Xue Yang, Xinghua Shao, Yue Sun, Wei Zhang, Xiaofang Li

**Affiliations:** School of Life Sciences, East China Normal University, Shanghai, China

**Keywords:** *Arabidopsis thaliana*, GAAP, ER stress, IRE1, cell death, unfolded-protein response

## Abstract

The function of human Golgi antiapoptotic proteins (GAAPs) resembles that of BAX inhibitor-1, with apoptosis inhibition triggered by intrinsic and extrinsic stimuli. However, little is known about the function of GAAP-related proteins in plants. Here, we studied Arabidopsis GAAP1 and GAAP3 and found that they were localized on the cellular membrane, including the endoplasmic reticulum (ER) membrane. The function of GAAP1/GAAP3 in ER-stress response was tested, and results showed that single or double mutation in GAAP1 and GAAP3 reduced plant survival and enhanced cell death under ER stress. The expression of both genes was induced by various abiotic stress signals. Quantitative real-time polymerase chain reaction analysis showed that GAAP1/GAAP3 level affected the expression pattern of the unfolded-protein response (UPR) signaling pathway genes upon prolonged ER stress. The mutation in both GAAP1 and GAAP3 genes promoted and enhanced UPR signaling when confronted with mild ER stress. Moreover, GAAP1/GAAP3 inhibited cell death caused by ER stress and promoted plant-growth recovery by turning down inositol-requiring enzyme 1 (IRE1) signaling after ER stress had been relieved. Co-immunoprecipitation (Co-Ip) and BiFC assays showed that GAAP1/GAAP3 interacted with IRE1. These data suggested that GAAP1/GAAP3 played dual roles in the negative regulation of IRE1 activity and anti-programmed cell death.

## Introduction

The endoplasmic reticulum (ER) is highly sensitive to physiological conditions or environmental stimuli. ER stress generally occurs when unfolded or misfolded proteins aggregate or when the load of client proteins exceed the folding capacity of the ER, which is caused by many adverse abiotic and biotic conditions. Pharmacologic agents such as tunicamycin (TM), an inhibitor of *N*-linked glycosylation, and dithiothreitol (DTT), a redox reagent, are widely used to induce ER stress in the laboratory (Yang et al., 2016). When facing ER stress, cells activate the unfolded-protein response (UPR) to address the problem. In mammalian cells, three UPR signaling pathways are initiated by ER transmembrane receptors, namely, inositol-requiring enzyme 1 (IRE1), protein kinase RNA-activated-like ER kinase (PERK), and activating transcription factor 6 (ATF6), to promote cell survival by restoring ER homeostasis (Walter and Ron, 2011; Brewer, 2014). Except for PERK ortholog, UPR pathways mediated by IRE1 and ATF6 homologs have been identified in plants (e.g., the IRE1 and bZIP28 pathways in Arabidopsis) (Liu et al., 2007; Deng et al., 2011; Nagashima et al., 2011; Humbert et al., 2012). IRE1 acts by splicing messenger RNA encoding transcription factor XBP1 in mammalian cell or bZIP60 in plant cell, respectively, to upregulate genes encoding factors that aid in protein folding and degradation (Nagashima et al., 2011; Iwata and Koizumi, 2012). Activated IRE1 also cleaves and degrades some mRNAs that reduce protein loading in the ER (Mishiba et al., 2013). NAC103 has been identified as the direct target of the spliced bZIP60 and relays ER stress signals to UPR downstream genes (Sun et al., 2013). Similar to ATF6, Arabidopsis bZIP28 transports to the Golgi and undergoes regulated intramembrane proteolysis before moving into the nucleus to upregulate the expression of genes involved in various ER quality-control processes under ER stress (Haze et al., 1999; Liu et al., 2007; Walter and Ron, 2011). However, if ER stress is severe and the cytoprotective outcomes are insufficient to restore ER homeostasis, the UPR triggers cell-death program to kill ER stress cells (Ogawa and Mori, 2004; Lin et al., 2007; Dai et al., 2016). In mammalian cells, IRE1 exhibits different activities or functions depending on various partner proteins. For example, the association of the BCL-2 family members BAX and BAK with the cytosolic domain of IRE1α regulates the initiation and duration of IRE1α adaptive activity (Hetz and Glimcher, 2009; Woehlbier and Hetz, 2011; Hetz, 2012). Meanwhile, BAX inhibitor-1 (BI-1) interacts with IRE1α and negatively regulates its activity (Lisbona et al., 2009). Under irreversible ER stress, IRE1α interacts with tumor necrosis factor receptor-associated factor 2 to promote cell death (Urano et al., 2000; Dhanasekaran and Reddy, 2008; Brewer, 2014; Zheng et al., 2017). Diminished IRE1α activity, concomitant with ongoing PERK signaling, during prolonged ER stress may be pivotal in shifting the UPR toward a proapoptotic outcome (Lin et al., 2007, 2009). A growing body of evidence indicates that ER stress initiates UPR for cell survival and facilitates programmed cell death (PCD) under severe stress in plant. In Arabidopsis, the membrane transcription factor NAC089 is upregulated by bZIP28 and bZIP60 and activates downstream genes involved in PCD (Liu et al., 2007; Liu and Howell, 2010; Yang et al., 2014). However, little is known about how the activities of ER-stress sensors are regulated and how the different outcomes between cell survival and death effects can be determined in plants.

Genes that control PCD are conserved across wide evolutionary distances in metazoans (Lam, 2004). In mammals, ER-stress-induced cell death is a process controlled by the balance among various anti- and proapoptotic members of the BCL-2 protein family and antiapoptotic members of the BI-1 family (Lisbona et al., 2009; Flusberg and Sorger, 2015). Only BI-1 genes have been identified in plants to date. Similar to its homolog in mammals, plant BI-1 reportedly plays an important role as a survival factor under multiple stress conditions. However, plant BI-1 has little effect on UPR signaling under ER stress (Watanabe and Lam, 2008). Arabidopsis BI-1 has also been observed to attenuate the prosurvival function of bZIP28 during recovery from ER stress. Differently from animal cells, Arabidopsis BI-1 does not temper the ribonuclease activity of IRE1 under temporary ER stress (Ruberti et al., 2018). Six other BI-1-like proteins have been described, namely, transmembrane BAX inhibitor motif containing (TMBIM) 1–6 and 1b, with BI-1 being TMBIM6 in mammals. Five of TMBIM members are also denoted together as Lifeguard (LFG) family according to a phylogenetic analysis (Hu et al., 2009; Carrara et al., 2012, 2017). TMBIM4 (LFG4), also known as Golgi antiapoptotic proteins (GAAPs) are highly conserved throughout eukaryotes (Gubser et al., 2007; Carrara et al., 2017). TMBIM4 was first identified in humans as h-GAAP, which most closely resembles BI-1, with apoptosis inhibition triggered by intrinsic and extrinsic stimuli. *Arabidopsis thaliana* GAAP1 (AT4G14730) was predicted to encode GAAP-related proteins through BLAST searches with h-GAAP (Gubser et al., 2007). Five GAAP gene members are found in Arabidopsis, and they are named AtLFG1-5 in another report. GAAP1/LFG1 and GAAP2 (At3g63310)/LFG2 proteins are further reportedly involved in modulating the interaction between plant and biotrophic powdery mildew fungi (Weis et al., 2013). However, whether Arabidopsis GAAPs have an evolutionarily conserved function in regulating PCD induced by abiotic stimuli remains unknown.

Here, we analyzed the cellular localization of GAAP1 and GAAP3 (At4g02690) in plant cells and their expression patterns. The two proteins resided on cellular membrane, including ER membrane. Both genes were expressed at very low levels during the seedling stage and induced by various stress signals. We found that GAAP1 and GAAP3 levels were critical to plant survival under ER stress. Molecular analysis further showed that the ectopic expression of GAAP1/GAAP3 delayed UPR activation, whereas mutation in both genes promoted and enhanced UPR signaling when confronted with mild ER stress. Moreover, GAAP1/GAAP3 inhibited cell death induced by ER stress and promoted plant-growth recovery by turning down UPR process mediated by IRE1 after the ER stress had been relieved. Co-immunoprecipitation (Co-IP) and BiFC assays showed that GAAP1/GAAP3 interacted with IRE1. All these data suggested that GAAP1/GAAP3 played dual roles in the regulation of UPR and PCD.

## Materials and Methods

### Plant Material and Growth Conditions

*Arabidopsis thaliana* of ecotype Columbia-0 (*Col*) plants and T-DNA insertion mutants in the Col-0 background were used. The mutants *gaap1-1* (Salk_046652c), *gaap1-2*(CS814417), and *gaap3* (SALK_001992) were isolated from the Salk T-DNA collection. The T-DNA insertion site was confirmed by the polymerase chain reaction (PCR) amplification of plant genomic DNA with T-DNA primers and gene-specific primers. The double mutant *gaap1gaap3* was generated by the hybridization of *gaap1-1* and *gaap3*. Seeds were stratified at 4°C for 2–3 days before germination, and plants were grown under continuous white light at 23 ± 2°C in soil or on 1/2 MS medium (1% sucrose, 0.8% agar).

To test the sensitivity of seedlings to ER stress, unless otherwise specified, 3-day-old seedlings grown on a filter paper placed on a 1/2 MS agar plate were transferred onto a plate containing different concentrations of TM or DTT for various times. To test growth recovery, 4-day-old seedlings that had been infiltrated with 1/2 MS liquid salt containing 0.00, 0.15, and 1.00 μg mL^-1^ TM for 6 h were transferred onto 1/2 MS solid medium. The fresh weight of seedlings was determined during recovery time, and inhibition rate was calculated by the decrement divided by the control weight. All calculations were performed using data from three independent experiments.

For RT or qRT assay, unless specifically noted, 7-day-old seedlings were incubated with 1/2 MS liquid medium containing different concentrations of TM for the times indicated. A medium containing 0.1% DMSO was used as control.

### Plasmid Construction

The open reading frame of *GAAP1* and *GAAP3* gene was introduced into the binary vector pMon530 (Monsanto, United States) under the 35S-promoter respectively to generate *GAAP1* and *GAAP3* overexpression constructs.

To illustrate the cellular localization of GAAP1 and GAAP3, the C-terminal of both gene fusion to YFP, named as 35S::GAAP1-YFP and 35S::GAAP3-YFP, were constructed in the binary pHB vector (Luo et al., 2014). The N-terminal of both gene fusions to YFP was constructed in pMon530 vector to generate YFP-GAAP1 and YFP-GAAP3, respectively. The HDEL sequence is the ER location signal (Srivastava et al., 2013), and the ER-marker CFP tags with HDEL signal was prepared by amplifying CFP-HDEL and ligating it into pMon530.

To make promoter-GUS (β-D-glucuronidase) constructs, genomic DNA sequences corresponding to 3600 bp upstream of the ATG codon of the GAAP1 ORF and 1041 bp promoter of GAAP3 were cloned into pBI101.1 vector, respectively.

For the BiFC assay, we fused the N- and C-terminal halves of YFP to the N-terminus of GAAP1/GAAP3 and C-terminus of IRE1A encoding the kinase and endoribonuclease domains, respectively. The vectors of BiFC (pXY104 and pXY106) were used.

The primers pairs are listed in Supplementary Table S1, and all generated constructs were confirmed by sequencing.

### Plant Transformation and Transgenic Plant Analysis

*Agrobacterium tumefaciens* strain GV3101 was used, and transformation was performed using the floral-dip method. The phenotypic effects of *GAAP1* and *GAAP3* in transgenic Col plants were analyzed in more than five independent transgenic lines. Transgenic plants carrying GAAP1::GUS and GAAP3::GUS were further selected for GUS activity detection as previously described (Li et al., 2009). Seedlings of GAAP1::GUS and GAAP3::GUS plants were incubated for 12 and 6 h, respectively at 37°C in the staining buffer. The inflorescence organs of GAAP1::GUS and GAAP3::GUS were incubated 24 and for 12 h respectively.

Transient-expression experiments were performed as previously described (Sparkes et al., 2006). The tag-fusion constructs were introduced into tobacco (*Nicotiana clevelandii*) leaf epidermal cells using the *Agrobacterium tumefaciens*-mediated infiltration technique.

### Protein Subcellular Localization and BiFC Assays

Subcellular localization of the fluorescent proteins was determined by confocal laser scanning microscopy (Leica TCS SP5II). Fluorescing cells were imaged using a filter set with excitation wavelengths of 510 and 436 nm, as well as emission filters at 517–540 and 488–490 nm, for YFP fusion and CFP fusion, respectively. For FM4-64 staining, roots of 4-day-old vertically grown seedlings were used and performed as previously reported (Bolte et al., 2004). Protein colocalization and BiFC assays were as previously described (Luo et al., 2014).

### Histochemistry and Microscopy

Propidium iodide (PI) and fluorescein diacetate (FDA, Sigma-Aldrich) staining as fluorescent indicators of cell-membrane permeability and cell viability, respectively, were performed as previously described (Watanabe and Lam, 2008). Nuclei in root cells were stained using 4′,6-diamidino-2-phenylindole (DAPI; Sigma-Aldrich) at 0.5 μg mL^-1^ in 0.1% (v/v) Triton X-100 for 10 min and then washed twice with water. DAPI-stained nuclei were observed under a fluorescence microscope (excitation = 390 nm; emission = 460 nm). Cell death detection with trypan blue staining was performed as described (Duan et al., 2010; Leite et al., 1999; Ning et al., 2002). H_2_O_2_ was detected with an endogenous-peroxidase-dependent *in situ* histochemical staining procedure using 3,3′-diaminobenzidine (DAB) (Thordal-Christensen et al., 1997). Four to five biological replicates were conducted for each staining, and at least 20 samples were determined for each genotype every replicate.

### Ion Leakage Measurement

The progression of cell death was assayed by measuring ion leakage from shoots after TM treatment. For each measurement, 20 shoots were immersed in 10 mL of distilled water with gentle shaking for 2 h at room temperature. The conductivity of the bathing solution was directly measured with a conductivity meter (METTLER TOLEDO SevenCompact S230). Measurements for each sample were performed at least in triplicate.

### Quantitative Real-Time Reverse-Transcription PCR (qPCR)

Total RNA from different tissues was extracted from a frozen tissue using TRIZOL reagent (Invitrogen), and the first cDNA strand was generated according to the instructions for Superscript RT (Toyobo, Japan). qPCR analysis was performed with three to six independent biological replicates, and data were analyzed as previously reported (Schmittgen and Livak, 2008; Li et al., 2013). The relative UPR gene expression was the expression level of each gene in different genotype plants normalized to the level in the wild-type control, both of which were normalized to the expression of *ACTIN8*. The specific primers for each gene are listed in Supplementary Table S1. Two-way ANOVA was performed, and Tukey’s range (honestly significant difference) test was used to determine significant differences among genotypes.

### Co-IP of Interacting Proteins

For the Co-IP assay, the truncated form of IRE1A containing kinase and RNase domains (amino acids 375–881) was cloned into pCAMBIA1300-35S-X-TAP plasmid (Li et al., 2012) to produce a TAP fusion construct, IRE1A-KR-TAP. FLAG-epitope-tagged GAAP3 cDNA was cloned into pCAMBIA1300-35S-3 × FLAG vector (Li et al., 2012) at *Bam*HI and *Sal*I sites respectively. IRE1A-KR-TAP and FLAG-GAAP3 were co-transformed into tobacco leaves. Leaves about 2–3 days after transformation were ground in liquid nitrogen, and proteins were isolated as previously described (Iwata et al., 2008). We used anti-Flag M2 affinity gel (Sigma) to capture FLAG-tagged proteins following the manufacturer’s instruction. Western blot was performed using the anti-FLAG M2 antibody and peroxidase–antiperoxidase soluble complex (Sigma).

## Results

### GAAP1 and GAAP3 Protein Localized on Membrane Including ER Membrane

**FIGURE 1:**
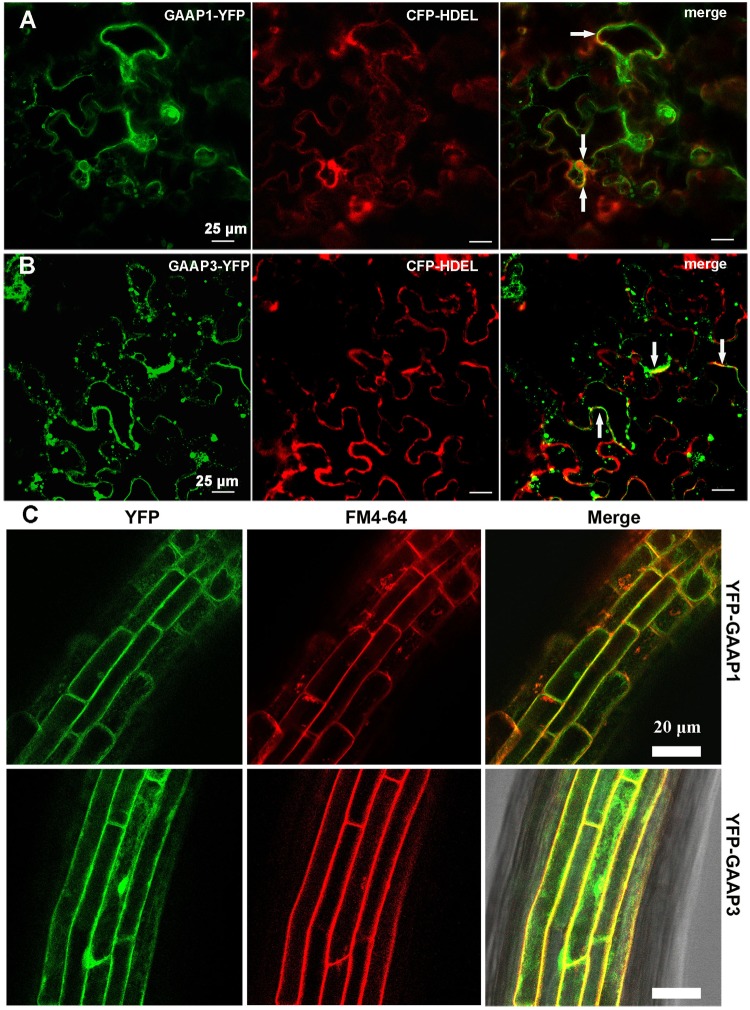
GAAP1 and GAAP3 are located on cellular membrane including the ER.Observation of the fluorescence of GAAP1-YFP **(A)** and GAAP3-YFP **(B)** co-transformed with ER marker CFP-HDEL in tobacco leaf epidermal cells. Yellow signals represent co-localization of two proteins, which are pointed by arrows. **(C)** Fluorescence of YFP-GAAP1/GAAP3 merged with the membrane marker FM4-64 in the root cells of stable Arabidopsis transformants.

GAAP1 and GAAP3 are closely related proteins sharing strikingly similar sequences (53.4% identity and 76.1% similarity). Similar to the secondary structure of mammalian GAAP protein, Arabidopsis GAAP1 and GAAP3 protein were also supposed to have seven transmembrane domains according to the prediction system^1^. To determine the subcellular localization of GAAP1 and GAAP3, the ER marker CFP-HDEL was co-transformed with GAAP1–YFP or GAAP3–YFP in tobacco epidermal cells. The fluorescence signal of both GAAP1–YFP and GAAP3–YFP were found to be close to the membrane and cytoplasm. At least part of the fluorescence signal of both GAAP1–YFP and GAAP3–YFP was co-localization with the ER marker CFP-HDEL (**Figures 1A,B**). Additionally, the stable transgenic Arabidopsis plants transformed with YFP fused with the N-terminal of GAAP1 and GAAP3 constructors were obtained. Most of the fluorescence signals of YFP–GAAP1 or YFP–GAAP3 merged with FM4-64 dye in root cells (**Figure 1C**). FM4-64 is a membrane-selective fluorescent dye frequently used as a membrane and endosome marker (Bolte et al., 2004; Zonia and Munnik, 2008). These data suggested that both proteins were located on cellular membrane including ER membrane.

### GAAP1 and GAAP3 Preferentially Expressed in the Reproductive Organs

**FIGURE 2:**
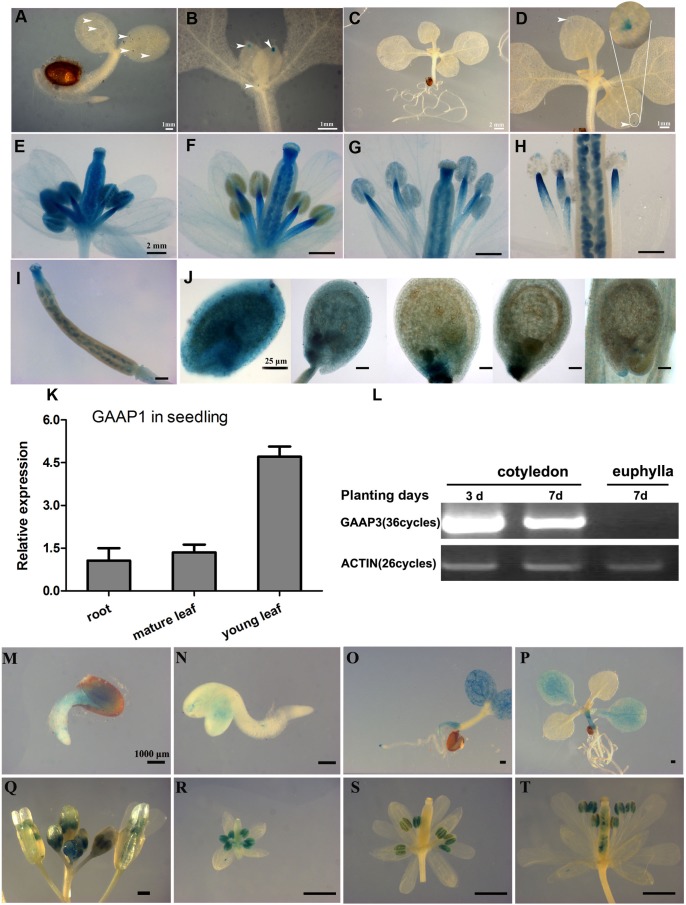
Expression patterns of *GAAP1* and *GAAP3* in Arabidopsis. GUS staining of *GAAP1* in 3-day-old seedling **(A)**, in the above-ground area of 7-day-old seedling **(B)**, in a 10-day-old seedling **(C,D)**, in the development of the flower **(E–H)**, in a young silique **(I)**, and in the development of seeds **(J)**. The arrowhead indicates the staining signal, and the encircled region in **(D)** is magnified. Bars = 1 mm **(A,B,D)**, 2 mm **(C,E–I)**, and 25 μm **(J)**. **(K)** Expression pattern of *GAAP1* genes in 7-day-old seedlings assayed by q-PCR. Data are represented at the fold level relative to the level in the root. **(L)** Expression pattern of *GAAP3* genes in young seedlings assayed by RT-PCR. GUS activity of *GAAP3* during seed germination **(M,N)**, in 4- and 14-day-old seedlings **(O,P)**, in the whole inflorescence **(Q)**, in the development of flower at stage 7–9 **(R)**, stage 10–12 **(S)**, and stage 13 **(T)**.

To illustrate the expression patterns of *GAAP1* and *GAAP3*, GUS staining in GAAP1/GAAP3::GUS transgenic Arabidopsis plants in addition to qRT-PCR or RT-PCR assay was performed. Results showed that *GAAP1* was weakly expressed in young seedlings and exclusively expressed in the reproductive organs (**Figures 2A–K**). *GAAP1* was expressed mainly at the tip of leaf primordium and young leaf during the seedling stage (**Figures 2A–D,K**). GAAP1::GUS activity and transcripts level were observed to be higher in flower buds and young siliques (**Figures 2E–J**). In flower bud, GUS signal was observed in the stamen and pistil (**Figure 2E**). With flower development, no GUS staining was found in the full-grown anther, but in the filament (**Figures 2F–H**). GUS activity was strong in the ovule during female megagameto genesis. With the fertilization onset, signals began to decrease from the chalazal pole to the micropylar pole. GUS staining was concentrated around the embryo and micropylar endosperm at the globular stage of embryo. By the time of the heart stage, signals were observed only at the site of micropylar pole (**Figure 2J**).

*GAAP3* signal appeared during seed germination and was mainly expressed in the cotyledon and the root during the young-seedling stage (**Figures 2L–N**). *GAAP3* expression in cotyledon weakened with seedling growth (**Figures 2L,O,P**). *GAAP3* was highly expressed throughout the entire developmental stage of the anther and in the style after pollination in addition to silique (**Figures 2Q–T**).

### *GAAP1* and *GAAP3* Expression Levels Were Induced by Several Stress Signals

**FIGURE 3:**
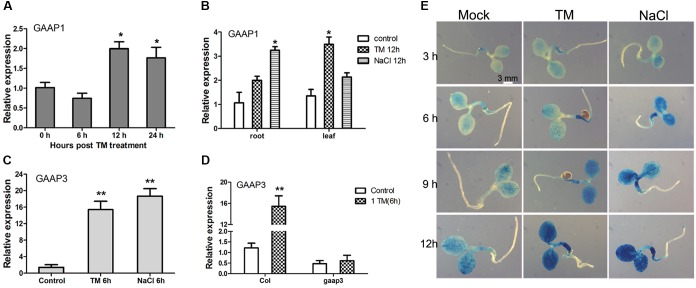
Expression levels of *GAAP1* and *GAAP3* genes were induced by stress signals in Arabidopsis seedling. **(A)** Expression level of *GAAP1* in 10-day-old plants in the presence of 0.5 μg mL^-1^ TM at different hours, as assayed by q-PCR. **(B)** Expression level of *GAAP1* in the root or leaf of 10-day-old plants in the absence or presence of 0.5 μg mL^-1^ TM or 100 mmol L^-1^ NaCl for 12 h, as assayed by q-PCR. **(C)** Expression level of *GAAP3* in 4-day-old plants in the presence of 0.5 μg mL^-1^ TM or 100 mmol L^-1^ NaCl for 6 h, as assayed by q-PCR. **(D)** Expression level of *GAAP3* in 4-day-old seedlings of Col and *gaap3* in the absence or presence of 1.0 μg mL^-1^ TM for 6 h, as assayed by qPCR. The relative gene expression was the level normalized to the control level in the wild-type (Col). **(E)** GUS staining for *GAAP3*::GUS in a 3-day-old seedling treated without or with 100 mmol L^-1^ NaCl or 0.5 μg mL^-1^ TM for 3–12 h. Data are represented as mean ± SE for at least three biological replicates and the asterisk indicates significant differences between treatment and control samples (^∗^ *p* < 0.05, ^∗∗^ *p* < 0.01).

The effects of TM or salt treatment on *GAAP1* and *GAAP3* expression during the seedling stage were tested. As shown in **Figure 3A**, *GAAP1* transcripts did not change in seedlings before 6 h upon treatment with 0.5 μg mL^-1^ TM. The transcripts’ level increased but not significantly with the progression of ER stress over 12 h. Real-time PCR analysis also showed that the level of *GAAP1* transcript in roots and leaves was up-regulated by chronic treatment with TM and 100 mmol L^-1^ NaCl to some extent (**Figure 3B**). However, *GAAP3* transcripts were up-regulated quickly and significantly by ER stress and salt stress in cotyledons, hypocotyls, and root tips assayed by qPCR and promoter-GUS reporter assay (**Figures 3C–E**). Furthermore, *GAAP3* gene was not enhanced in *gaap3* mutant upon TM treatment (**Figure 3D**).

### Mutations of GAAP1 and GAAP3 Enhanced the Plant Sensitivity to ER Stress

**FIGURE 4:**
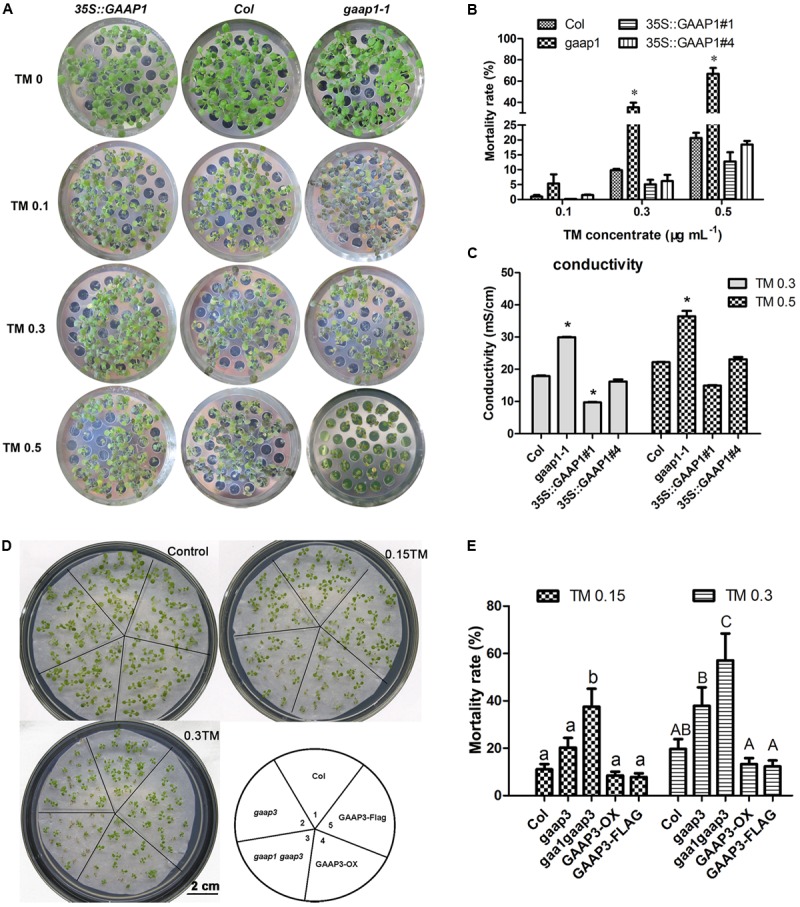
Mutation of GAAP1 and/or GAAP3 enhanced plants sensitivity toward TM. **(A–C)** GAAP1 mutation-enhanced plant death induced by TM. **(A)** Growth of Col, *35S::GAAP1#1* seedlings on 1/2 MS medium for 5 days, and then moved on 1/2 MS medium supplied with different concentrations (0–0.50 μg mL^-1^) of TM for 1 week. **(B)** Mortality rate of *GAAP1*-overexpressing transgenic plants, and Col and *gaap1-1* treated with different concentrations of TM for 1 week. Error bars depict SE of six independent experiments. Error bars depict SD. Significant differences compared with Col plants at the same concentrations of TM, as indicated by asterisks (*^∗^p* < 0.05, n > 80). **(C)** Damage of GAAP1-overexpressing transgenic plants, Col and *gaap1-1* treated with 0.3 and 0.50 μg mL^-1^ TM for 1 week. Shoots were collected from the samples obtained from the different sets of seedlings performed for **(B)** and then subjected to ion leakage measurements. Data are expressed as mean ± *SD*. Significant differences compared with Col plants at the same concentrations of TM, as indicated by asterisks (^∗^*p* < 0.05, *n* = 3). **(D,E)** *GAAP3* single mutation or GAAP3 and GAAP1 double mutations reduced the resistance of plants to ER stress. Phenotypes **(D)** and mortality rates **(E)** of Col, *gaap3, gaap1gaap3*, *GAAP3*-OX, and *GAAP3*-FLAG, which grew on 1/2 MS medium without or with 0.15 and 0.30 μg mL^-1^ TM for 10 days. Data are expressed as mean ± SE of five independent experiments. Different letters indicate significant differences between different plants with the same TM treatment subjected to χ^2^ test.

To determine whether GAAP1 and GAAP3 can resist ER stress, we obtained loss-of-function mutant *gaap1-1*, gene knock-down mutants *gaap1-2* and *gaap3*, and double mutant *gaap1-1 gaap3* in addition to transgenic lines overexpressing *GAAP1*or *GAAP3* in Col (Supplementary Figure S1). All mutants and transgenic plants did not exhibit obvious growth defects above ground when growing on soil under normal growth conditions. When seedlings were transferred to TM-containing (0.1–0.5 μg mL^-1^) medium to grow for 7–10 days, plant survival decreased in a TM-dose-dependent manner (**Figure 4**). In terms of mortality rate and membrane permeability indicated by electrical conductivity, GAAP1/3 mutation reduced plant survival subjected to ER stress. *GAAP1*or *GAAP3*-overexpressing seedlings survived the best upon chronic TM damage, although the mortality rates of the transgenic plants insignificantly differed from Col. ER stress was also triggered by DTT. Similarly, when 4-day-old seedlings were transferred into the medium with 5 mmol L^-1^ DTT, more severe growth inhibition was found earlier in *gaap1-1* and *gaap1gaap3* double mutants, and a higher death rate was observed in all mutants upon 5 mmol L^-1^ DTT for 10 days (Supplementary Figure S2). The double mutant *gaap1gaap3* was generally the most sensitive, and *gaap1-2* and *gaap3* were slightly more sensitive than the wild-type based on the rate of healthy plants and mortality (**Figure 4** and Supplementary Figure S2). To confirm that the T-DNA insertion in *gaap1-1* and *gaap3* was in fact responsible for the sensitivity defects, we transformed 35S::GAAP1 into *gaap1-1* mutant and GAAP3 driven by its native promoter into *gaap3* mutant respectively and found that the sensitivity of single mutant can be restored (data not shown).We also compared the primary root growth of each seedling on the medium containing TM. The root length of the *GAAP3*-overexpressing lines was less inhibited than that of the wild-type, whereas the primary root elongation of *gaap3* and *gaap1gaap3* double mutants was significantly impaired (Supplementary Figure S3). The inhibition of primary root growth of *GAAP1*-overexpressing line upon TM treatment was also reduced (data not shown). All these findings suggested GAAP1 and GAAP3 function in the resistance of plants to ER stress.

### GAAP1 and GAAP3 Functioned in Resistance to Cell Death Induced by ER Stress

**FIGURE 5:**
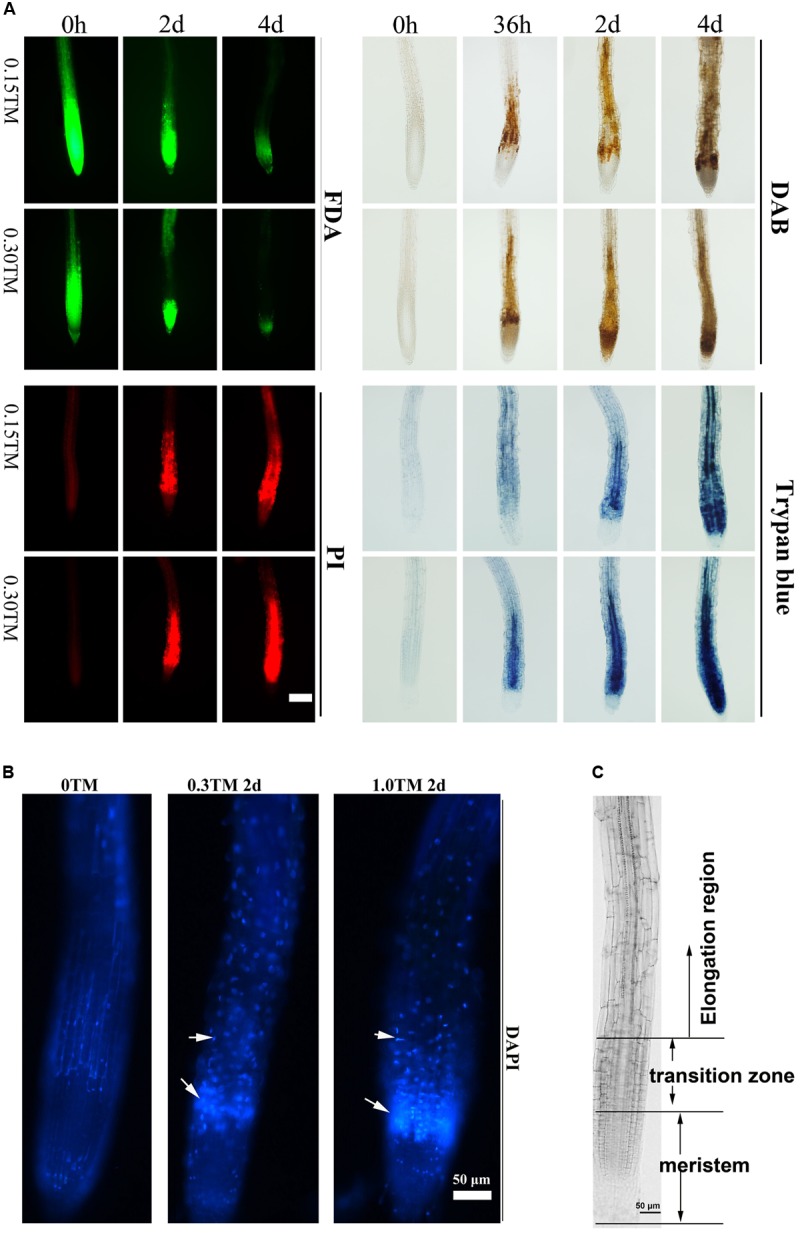
Root cell death pattern under ER stress. **(A)** Time course analysis of root cell viability and ROS level upon TM treatment. The 3-day-old seedlings of vertically cultivated Col were transferred to new culture plate containing different concentrations of TM (0, 0.15, and 0.30 μg mL^-1^) and root cells were stained by FDA, PI, DAB, and Trypan blue after 0 h, 36 h, 2 days, and 4 days, respectively. The staining level of root on the medium without TM did not change over the time course and were same as those at “0 h”. Bar = 100 μm. **(B)** Root cell death pattern stained by DAPI under ER stress. The 3-day-old seedlings of Col were transferred to a new liquid culture medium containing different concentrations of TM (0, 0.3, and 1.0 μg mL^-1^) for an additional 2 days and root cells were stained by DAPI. **(C)** Different regions of the primary root. Bar = 50 μm.

ER stress triggered by TM can induce PCD in rosette leaves and roots, and 0.30 μg mL^-1^ TM is reportedly the sublethal dose for plants (Watanabe and Lam, 2008; Mishiba et al., 2013). Three-day-old Col seedlings were transferred into medium containing 0, 0.15, and 0.30 μg mL^-1^ TM. The pattern of root cell of Col injured over the time course upon TM treatment was first examined by FDA, PI, DAB, and trypan blue staining to determine cell viability, cell membrane permeability, ROS level, and cell death, respectively (**Figure 5A**). At 36 h, the accumulation of H_2_O_2_ stained by DAB occurred between meristem and elongation region, and this area was denoted as the transition zone (**Figures 5A,C**) (Baluska et al., 2010). Moreover, cells of stele in transition zone decayed as showed by trypan blue staining only with 0.30 μg mL^-1^ TM, and much fewer cells died with 0.15 μg mL^-1^ TM. With further ER stress for 48 h, ROS accumulation extended to root meristem and elongation region, and cell death was enhanced with 0.30 μg mL^-1^ TM. Cell death in the transition zone also appeared with 0.15 μg mL^-1^ TM as showed by FDA, PI, and trypan blue staining (**Figure 5A**). DAPI is cell-membrane semipermeable, and fluorescence intensity significantly increases when they bind to nuclear DNA. With increased TM dose, root cells stained by DAPI also showed enhanced fluorescence signal in the transition zone (**Figure 5B**), which reflected increased cell-membrane permeability and condensed nuclei. Obviously, the cells in the transition zone were most sensitive to ER stress. With increased ER stress, ROS accumulation and cell death extended to root meristem and elongation region. The area of cell death increased in a TM-dose-dependent manner and over the time course.

**FIGURE 6:**
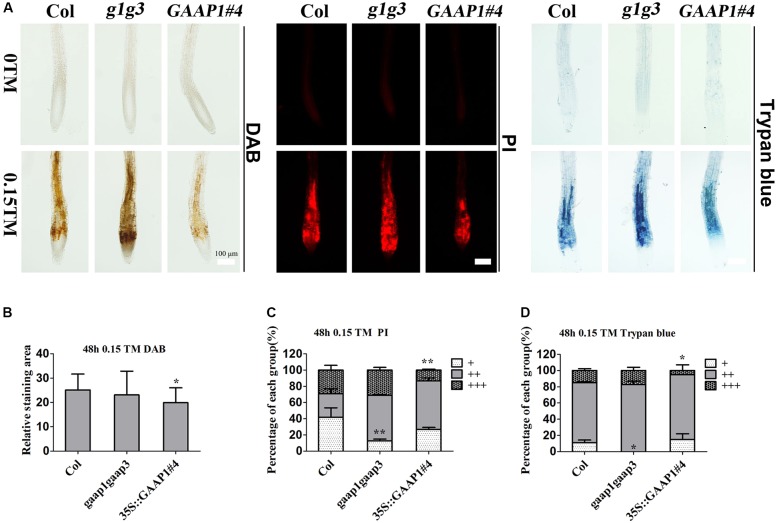
GAAP1/GAAP3 inhibited the cell death induced by ER stress. **(A)** The 3-day-old seedlings that were vertically cultured were transferred to a new culture plate containing different concentrations of TM. Root cells of Col, *gaap1gaap3* (g1g3), and 35S::GAAP1#4 treated without or with 0.15 μg mL^-1^ TM for 48 h were stained by DAB, PI, and trypan-blue. Bar = 100 μm. **(B–D)** The H_2_O_2_ level **(B)** and cell death severity **(C,D)** were analyzed by quantifying the staining degree. DAB staining area of the signal intensity more than a certain threshold was determined by ImageJ software. PI and trypan blue staining intensity were classified in three levels, namely, faint, medium, and strong, and depicted as “+”, “++”, and “+++”, respectively, according to Supplementary Figure S4. The percentage of each group was calculated. Error bars represent standard deviation. Significant differences compared with Col plants were indicated by asterisks (student test, ^∗^*p* < 0.05, *n* 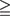 20).

To further determine whether GAAP1 and GAAP3 can inhibit PCD, we evaluated the root-cell viability of mutants and transgenic lines in response to TM for 48 h. The cells of *gaap1gaap3* double mutants showed the strongest signals, whereas 35S::GAAP1#4 and GAAP3-OX showed the weakest signals of DAB, PI, and trypan blue staining (**Figure 6** and Supplementary Figures S4, S5). These data suggested that GAAP1 and GAAP3 conferred increased tolerance to ER-stress-induced cell death.

### Effects of GAAP1 and/or GAAP3 Level on the Induction Pattern of UPR Genes Under Different ER Stress Conditions

**FIGURE 7:**
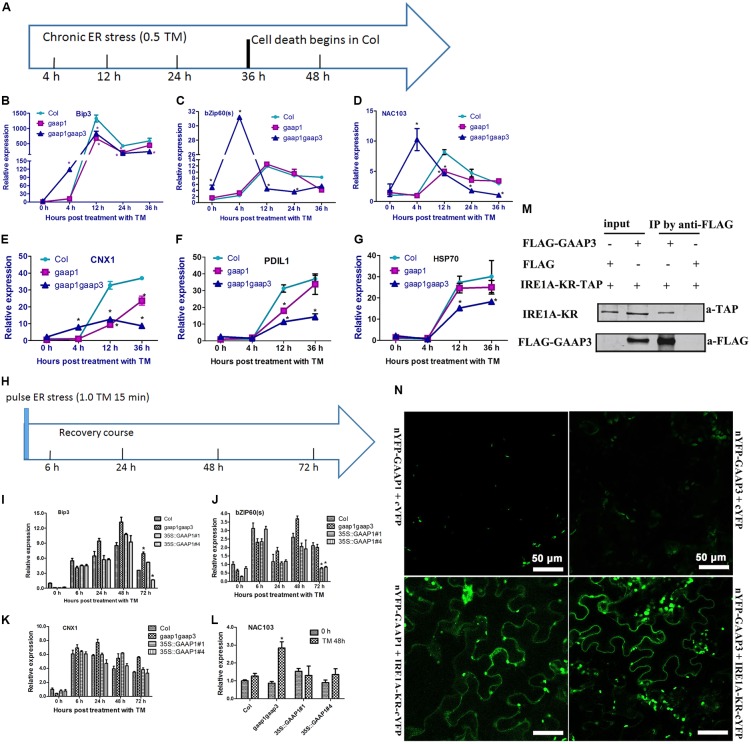
Expression levels of GAAP1 and GAAP3 affected the UPR gene pattern over the time course of chronic ER stress or during the recovery of ER stress through the association with IRE1. **(A–G)** The change pattern of UPR genes in Col, *gaap1-1*, and *gaap1gaap3* plants upon chronic ER stress induced by 0.5 μg mL^-1^ TM, and the treatment condition is shown in **(A)**. Transcript levels of selected ER marker genes were quantified by qRT–PCR. Value of each control of Col was set at 1. **(H–L)** GAAP1 enhanced the decline of UPR gene mRNA during ER stress recovery. Total RNA was isolated from 7-day-old Arabidopsis seedlings that were vacuum infiltrated with 1/2 MS liquid salt containing 1.0 μg mL^-1^ TM for 15 min and then recovered for 6, 24, 48, and 72 h on the medium, and the treatment condition is shown in **(H)**. Seedlings before vacuum infiltration with TM were used as 0 h. Transcript levels of selected ER marker genes were quantified by qRT–PCR. Value of each gene in Col at 0 h is set at 1. Data are from three to four biological replicates (± SD). Asterisks refer to significant differences from Col at the same time points (*p* < 0.05). **(M)** GAAP3 interacted with the region containing the kinase and endoribonuclease domains of Arabidopsis IRE1A (IRE1A-KR) in tobacco leaf cells assayed by Co-IP. **(N)** GAAP1 and GAAP3 interacted with IRE1A, as shown by BiFC assay. YFP fluorescence was observed when nYFP- GAAP1/GAAP3 and IRE1A-KR-cYFP were co-expressed in the tobacco leaf cell (bottom images), whereas only the background signal of chlorophyll was observed when nYFP- GAAP1/GAAP3 and cYFP were co-expressed (top images), or IRE1A-KR-cYFP and nYFP were co-expressed (not shown).

The hypersensitivity of *gaap1gaap3* mutants and the resistance of GAAP1 or GAAP3 overexpressing plants to ER stress prompted us to determine whether GAAP1/GAAP3 protein directly or indirectly interfered with the UPR. We performed real-time PCR analysis to examine the expression of markers for UPR activation under three ER stress conditions. To determine whether UPR signaling was directly disturbed, we performed an assay under acute ER stress induced by high-dose TM (5 μg mL^-1^) for 4–6 h. To examine the tunability of the UPR change pattern over the prolonged ER stress, we performed an assay under ER stress conditions caused by 0.5 μg mL^-1^ TM for 4–36 h, which caused cell death (**Figure 5**). To examine the tunability of the UPR change pattern upon pulse-ER stress, we performed an assay during the recovery course after the short ER stress (treatment with 1.0 μg mL^-1^ TM for 15 min), which did not inflict much cell damage. Upon acute ER stress, the induction values of *AtBIP3*, *AtBIP2*, spliced *AtbZIP60* (*bZIP60s*), *AtPDIL*, *AtCRT1*, and *AtCNX1* and *AtHSP70*, which are common markers for UPR activation (Martinez and Chrispeels, 2003; Williams et al., 2010), in Col, *gaap1-1*, *gaap1gaap3*, and the *GAAP1/GAAP3*-overexpressing plants were similar, except for the lower upregulation of *bZIP60s* in GAAP1-overexpressing line (Supplementary Figures S6, S7). Additionally, the expression patterns of UPR genes in Col, *gaap1-1*, and *gaap1gaap3* seedlings over the treatment course with 0.5 μg mL^-1^ TM were compared (**Figures 7A–G**). Genes *bZIP60s* and *NAC103* downstream the IRE1-dependent signaling pathway, genes *AtPDIL, AtCNX1*, and *AtHSP70* downstream the bZIP28-dependent signaling pathway, and *AtBIP3*, which belongs to both pathways, were selected (Liu et al., 2007; Mishiba et al., 2013). The data showed that the IRE1 pathway genes peaked and then declined with persistent ER stress, and the bZIP28 pathway genes remained upregulated over 36 h. *GAAP1* and *GAAP3* mutations enhanced *bZIP60s*, *NAC103*, and *AtCNX1* upregulation at 4 h following TM treatment. However, the expression levels and patterns of *bZIP60s* and *NAC103* downstream the IRE1-dependent signaling pathway were severely affected by the *gaap1gaap3* double mutant, thus peaking earlier and higher than Col following ER stress. The transcript levels of *bZIP60s* and *NAC103* displayed a decline with prolonged ER stress at around 36 h in Col and *gaap1-1* seedlings, and around 12 h in the *gaap1gaap3* double mutant (**Figures 7C,D**). Only the expression levels were different, but not the patterns of the bZIP28 pathway genes, in the mutants over the time course (**Figures 7B**,**E–G**). The upregulation of most UPR genes tested were reduced in the single *gaap1-1* and double *gaap1gaap3* mutants over 12 h following TM treatment. These data suggested that GAAP1 and GAAP3 play antagonistic roles in UPR gene induction at the beginning of mild ER stress. However, with persistent ER stress, the UPR gene induction level was lower in the *gaap1* single and *gaap1gaap3* double mutants than those in the wild-type. A previous report showed that TM at 0.5 μg mL^-1^ for 3 days is lethal for Arabidopsis seedlings (Watanabe and Lam, 2008). The above data showed that root cell death can be detected with 0.3 μg mL^-1^ TM for 36 h, and the cell death induced by TM was enhanced in *gaap1gaap3* (**Figures 5**, **6**). Accordingly, the attenuated induction of UPR gene mRNA in *gaap1* single and *gaap1gaap3* double mutants upon chronic ER stress induced by 0.5 μg mL^-1^ TM (**Figures 7A–G**) may be caused by the cell damage in mutants.

To avoid cell damage caused by high-dose or chronic ER stress, we evaluated the expression pattern of UPR genes in *gaap1gaap3* and *GAAP1*-overexpressing seedlings during the recovery course from short-term ER stress. **Figures 7H–K** show similar upregulation levels of representative UPR genes of both signaling pathways, including *AtBIP3*, *bZIP60s*, and *AtCNX1*, in all plant lines upon recovery for 6–48 h. By 72 h, the transcripts levels of *AtBip3* and *bZIP60s* decreased and both genes were nearly reduced to the basal level in the two *GAAP1*-overexpressing lines. *NAC103*, the direct target of *bZIP60s*, showed the highest level in *gaap1gaap3* mutant 48 h post recovery (**Figure 7L**). The levels of bZIP28 pathway marker gene *AtCNX1* in all plant lines were not different (**Figures 7G–J**). These data suggested that alterations in *GAAP1/GAAP3* expression levels may interfere with the UPR at the transcriptional level, and that GAAP1 and GAAP3 enhance the attenuation of the IRE1 signaling pathway activation during recovery from the ER stress fluctuation.

### GAAP1/GAAP3 Interacted With IRE1 *in Vivo*

The above results of the molecular pattern assay over the time course of ER stress suggest that the expression of GAAP1/GAAP3 negatively modulates the splicing activity of IRE1 for bZIP60. To test if GAAP1/GAAP3 regulates IRE1 directly, we searched for the interaction between GAAP3 and IRE1A. The region containing the kinase and endoribonuclease domains of Arabidopsis IRE1A fused with the N-terminal TAP tag was generated based on the result that BI-1 interaction requires the cytosolic C-terminal region of IRE1α encoding the kinase and endoribonuclease domains in the human cell (Lisbona et al., 2009). Co-IP experiments using lysates from tobacco leaf cells that were co-transformed with IRE1A-KR-TAP and FLAG-GAAP3 showed an association between both proteins (**Figure 7M**). For the BiFC assay, we fused the N- terminal halves of YFP to the N termini of GAAP1 and GAAP3, and the C-terminal halves of YFP to the C-termini of IRE1A and IRE1B, which encode the kinase and endoribonuclease domains, respectively. Strong YFP fluorescence was observed when the nYFP-GAAP1/GAAP3 and IRE1A-KR-cYFP or IRE1B-KR-cYFP were coexpressed in the tobacco leaf cell (**Figure 7N** and Supplementary Figure S8), suggesting that GAAP1/GAAP3 interacted with IRE1A or IRE1B.

### Overexpressing GAAP1 and/or GAAP3 Were Conducive to the Growth of Plants After the ER Stress

**FIGURE 8:**
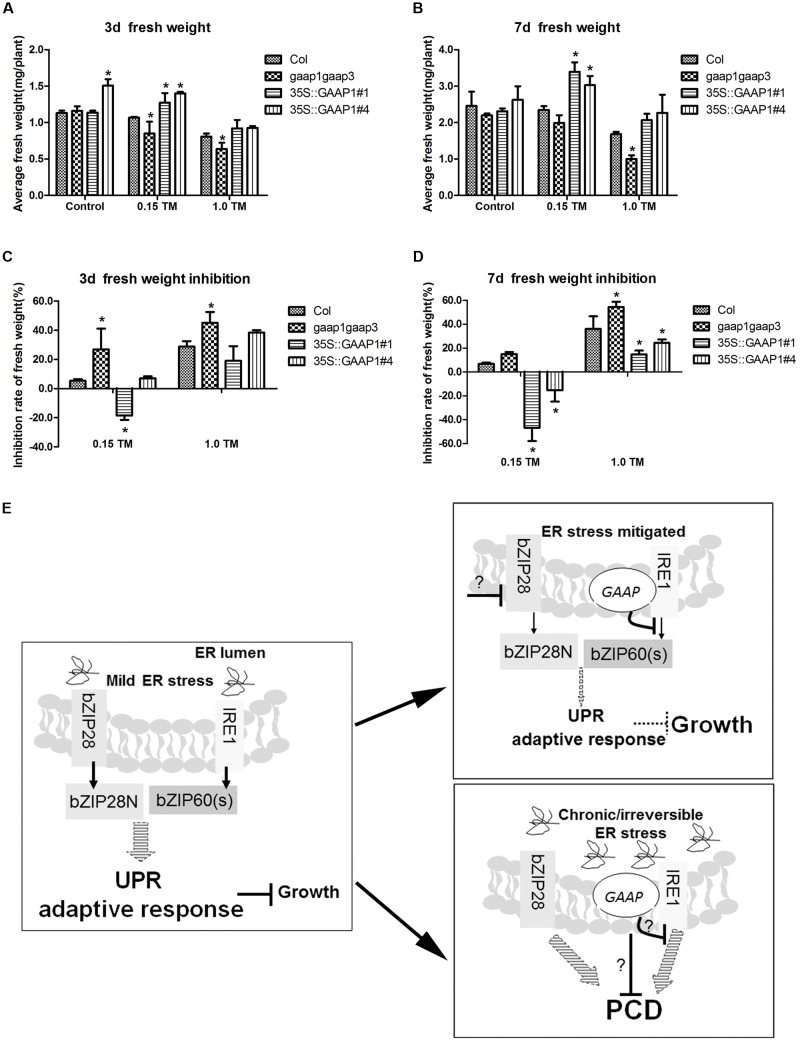
GAAP1/GAAP3 promoted growth recovery after mild ER stress was relieved and the working model of the function of GAAP1/GAAP3.**(A–D)** The 4-day-old seedlings that were infiltrated with 1/2 MS liquid salt containing 0, 0.15, and 1.0 μg mL^-1^ TM for 6 h were transferred to the 1/2 MS solid medium. Fresh weight of seedlings **(A,B)** and inhibition rates **(C,D)** were determined after recovery for 3 and 7 days. Data are from three biological replicates (± SE) and at least 30 samples for each plant line were used for each treatment. Asterisks refer to significant differences from Col at the same time points and same conditions (*t*-test; *p* < 0.05). **(E)** Working model of the dual role of GAAP1/GAAP3 in the regulation of UPR and PCD. In cells undergoing mild ER stress, rapid adaptive responses were initiated through UPR signaling mediated by bZIP28 and IRE1 at the cost of growth inhibition. Usually the weak inhibition role of the low level of GAAP1/GAAP3 *in vivo* under such condition makes cells increases the competence for UPR signaling. The artificial ectopic expression GAAP1/GAAP3 will postpone the activated UPR signaling. GAAP1/GAAP3 level will be upregulated upon ER stress and then down-regulated upon UPR signaling by inhibiting IRE1 activity to soon recover plant growth when the ER stress is mitigated. GAAP1/GAAP3 negatively controls IRE1 activity via direct association. If the ER stress is persistent or severe, PCD will be induced. GAAP1/GAAP3 might regulate the downstream PCD machinery or negatively control IRE1, thereby inhibiting cell death initiated by diverse intrinsic death stimuli, including irreversible ER stress.

The results that GAAP1 and GAAP3 enhanced the attenuation of the IRE1 signaling pathway during the recovery from ER stress prompted us to further examine whether they are conducive to growth under such condition. The 4-day-old seedlings were infiltrated with 0, 0.15, and 1.00 μg mL^-1^ TM for 6 h and then transferred to the normal solid medium. No obvious etiolation or growth damage in all tested lines was observed during the culture. Moreover, the decreased fresh seedling weight of GAAP1 or GAAP3-overexpressing lines was significantly lower than that of the wild-type, whereas the inhibition rate of the *gaap1gaap3* seedling was higher (**Figures 8A–D** and Supplementary Figure S9). These data were consistent with the hypothesis that GAAP1 and GAAP3 promoted the down-regulation of the cell protective response and favored the energy redistribution for growth after mild ER stress was relieved.

## Discussion

### GAAP1 and GAAP3 Inhibit Cell Death Under ER Stress

GAAPs are broadly conserved cytoprotective proteins and they are localized in the membranes of the ER and the Golgi apparatus (Henke et al., 2011). Moreover, the human hGAAP inhibits apoptosis triggered by intrinsic and extrinsic stimuli (Gubser et al., 2007). GAAP1 and GAAP3, the homologs of h-GAAP in *Arabidopsis*, were localized on membranes, including the ER membrane, according to the protein secondary structure prediction and cellular localization assay (**Figure 1**). Consistent with this, GAAP1 and GAAP3 interacted with IRE1 (**Figures 7M,N** and Supplementary Figure S8), which is a UPR sensor located on the ER membrane. ER is the best-known organelle, aside from mitochondrion, for regulating the PCD in plant cells (Chen et al., 2010). Additionally, Arabidopsis *GAAP1* and *GAAP3* genes expression were enhanced, especially the *GAAP3* gene, under ER or salt stress conditions (**Figure 3**), which suggested that GAAP1 and GAAP3 are physiologically associated with cell death control and/or stress management. Moreover, TM disturbs root development in a dose-dependent manner, concomitantly with the loss of cell viability and induction of PCD phenotypes (Watanabe and Lam, 2008; Nagashima et al., 2011). In this study, the cells of the transition region of the root were the most sensitive to ER stress, and the scope and amount of cell death expanded and increased around this region with the severity of ER stress (**Figure 5**). Consistent with this, cells in the transition zone undergo notable cell wall alterations and display unique cytological and metabolic properties that allow them to sense and respond to diverse environmental factors and endogenous cues (Verbelen et al., 2006). The hypersensitivity of GAAP1 and GAAP3 single or double mutants and the resistance of plants that overexpressed *GAAP1* or *GAAP3* to ER stress (**Figures 4**, **6** and Supplementary Figures S3, S4) suggested that GAAP1 and GAAP3 play redundant roles in maintaining plant growth and survival under ER stress, at least partly by attenuating cell death. It has been shown that ER stress can induce ROS production and also oxidative stress can induce ER stress (Ozgur et al., 2014, 2015). ROS can be stress response signaling and also leads to cell death depending on its dose. GAAP1 and GAAP3 predominately located in the plasma membrane and affected ROS level upon ER stress (**Figures 1**, **6**). Whether GAAP1 and GAAP3 resistance to cell death induced by ER stress is mediated by the influence of ROS production needs further research.

### GAAP1/GAAP3 Inhibited IRE1 Pathway Under Mild ER Stress

IRE1A/B and bZIP28 are the two main pathways of UPR for the adaption in plants (Liu et al., 2007; Mishiba et al., 2013). Moreover, unmitigated ER stress induces PCD in animals and plants (Lisbona et al., 2009; Yang et al., 2014). However, knowledge regarding the different activities of UPR sensors between adaption and cell death stations in plant is limited. And little is also known how the UPR activation is down-regulated to ensure plant growth when the stress is mitigated. Recently, Arabidopsis BI-1 has been reported to attenuate the pro-survival function of bZIP28 but not temper the ribonuclease activity of IRE1 in recovery roles from temporary ER stress (Ruberti et al., 2018). To define the possible regulation of both pathways by GAAP1/GAAP3, we determined the level and pattern of the representative marker genes in the *gaap1gaap3* mutant over the time course of persistent ER stress. As expected, the protective marker genes of both UPR pathways were significantly upregulated upon 0.5 μg mL^-1^ TM treatment in all plant lines for several hours. Moreover, *gaap1gaap3* displayed a more rapid and more pronounced upregulation of UPR genes mRNA at the beginning of treatment, but lower induction level of UPR genes as ER stress proceeded, compared with Col. Additionally, the upregulation of the IRE1 pathway genes (*BIP3*, spliced *bZIP60*, and *NAC103*) declined with prolonged ER stress in all plants. In human cells, the activation IRE1 signaling pathway initially declines after prolonged ER stress, accompanied by cell death. Furthermore, cell survival can be enhanced if IRE1 activity is artificially sustained (Lin et al., 2007). In fact, cell death was observed around 36 h post treatment with 0.5 μg mL^-1^ TM (Watanabe and Lam, 2008) (**Figure 5**), the dose used to induce chronic ER stress. Cells in *gaap1gaap3* seedlings exhibited pronounced death upon chronic ER stress (**Figure 6** and Supplementary Figure S5). Moreover, the induction of IRE1 pathway genes peaked and weakened rapidly in the *gaap1 gaap3* plants (**Figures 7A–G**). A certain relationship may exist between the amplitude modulation of IRE1 activity and cell death. IRE1 mutation in plant or human cell enhances programmed cell death (Mishiba et al., 2013). These data suggest that plant and mammalian cells share the conserved regulation mechanism, which is the declining of the IRE1 adaptive pathway accompanied with the initiation of cell death secondary to persistent ER stress. Enhanced activation of the IRE1 pathway only upon the early stage of mild ER stress in *gaap1gaap3* seedlings (**Figures 7A–D**) and no significantly different induction levels in most UPR genes except for bZIP60(S) were observed when plants were treated with high doses of TM, namely, 5 μg mL^-1^, for a few hours (Supplementary Figures S6, S7), indicating that GAAP1/GAAP3 is a modulator of UPR and might function as the inhibitor of IRE1 adaptive pathway.

### GAAP1/GAAP3 Promoted the Growth Recovery After the Mild ER Stress Was Relieved by Weakening the UPR Activity

To further validate the possible inhibitory effects of GAAP1/GAAP3 on UPR, we monitored UPR genes in Col-, *gaap1gaap3*-, and *GAAP1*-overexpressing lines during the recovery period after the pulse TM treatment. Under such experimental conditions, no obvious plant death and etiolation were observed, and a similar upregulation level of representative UPR genes of both signaling pathways in all plant lines recovery for 6 h till 48 h. At 72 h post-recovery, the transcripts levels of *AtBIP3* and *bZIP60s*, which are IRE1A/B pathway genes decreased. Moreover, the IRE1A/B pathway genes nearly decreased the basal level in the GAAP1-overexpressing lines, whereas the highest level of *NAC103* gene was retained in the *gaap1gaap3* mutant at 48 h. The bZIP28 pathway-marker gene *AtCNX1* levels were not different in all plant lines at each time point (**Figures 7H–K**). These data showed that GAAP1 and/or GAAP3 might specifically enhance the attenuation of the IRE1 signaling pathway activation during the recovery from ER stress fluctuation. Moreover, engagement of the IRE1 pathway confers protection against ER stress. The inhibitory effect on UPR was evident when confronted with low doses of ER stressors, which resembled *in vivo* normal conditions, wherein plant cells are equipped to cope with injury (adaptive conditions). In agreement with these findings, we observed low levels of *GAAP3* and *GAAP1* and their expression level increased upon ER stress (**Figures 2**, **3**). Similar functions of the BI-1 upon inactivation of IRE1α signaling through interacting with each other in human cells have been reported (Lisbona et al., 2009). Additionally, GAAP1 and GAAP3 interacted with IRE1A or IRE1B in plants (**Figures 7M,N** and Supplementary Figure S8). The attenuation of the cytoprotective action of IRE1 signaling by GAAP1 and/or GAAP3 when the ER stress is relieved should be advantageous for normal growth recovery with sufficient energy. In agreement with such hypothesis, the improved biomass production in GAAP1- or GAAP3-overexpressing lines was determined after short-term ER stress (**Figures 8A–D** and Supplementary Figure S9). Thus, the results indicated that GAAP1/GAAP3 displays a dual function in fine tuning UPR signaling and downstream PCD (model in **Figure 8E**). Considering the low level of both genes in normal growth conditions and their upregulated expression levels secondary to stress signals, the primary functions of GAAP1/GAAP3 might be to weaken the UPR activity and make cells recover from protection to growth upon relief of ER stress. Along with the recent finding that Arabidopsis BI-1 attenuates the pro-survival function of bZIP28 in ER stress resolution, this finding indicates that BI-1-relevant members might be involved in fine tuning the activity of UPR receptors in plants. Therefore, the mechanism of the anti-PCD activity of GAAP1 and/or GAAP3 upon ER stress needs further research.

## Accession Numbers

The Arabidopsis Genome Initiative accession numbers for the proteins referred to in the paper are At4G14730 (GAAP1), At4G02690 (GAAP3), At3G10800 (bZIP28), At1g49240 (ACTIN8), At1G09080 (BIP3), At1G42990 (bZIP60), At5G24360 (IRE1B), At4G16660 (HSP70), At4g24190 (SHD), At5g61790 (CNX1), At5G64060 (NAC103), and At2G17520 (IRE1A).

## Author Contributions

KG and WF performed the assay about plant sensitivity to ER stress. WeiW performed the protein interaction and cellular localization assay. ZW, MZ, and XT conducted the UPR assay. WenW, XY, and XS performed the expression of GAAP1 and GAAP3. YS and WZ supervised the physiological experiments and participated in interpreting the morpho-physiological data. XL designed the experiments, supervised the study, analyzed the data, and wrote the paper.

## Conflict of Interest Statement

The authors declare that the research was conducted in the absence of any commercial or financial relationships that could be construed as a potential conflict of interest.The reviewer SHH and handling Editor declared their shared affiliation.

## Footnotes

1 http://harrier.nagahama-i-bio.ac.jp/sosui/sosui_submit.html

## Abbreviations

Bax: Bcl-2 associated X protein
BiFC: bimolecular fluorescence complementation
DAB, 3: 3′-diaminobenzidine
DTT: dithiothreitol
ER: endoplasmic reticulum
FDA: fluorescein diacetate
GUS: beta-D-glucuronidase
PCD: programmed cell death
PI: propidium iodide
qRT-PCR: quantitative real-time PCR
ROS: reactive oxygen species
TM: tunicamycin
UPR: unfolded protein response.
