# Supplementary material for: Arabidopsis GAAP1 and GAAP3 Modulate the Unfolded Protein Response and the Onset of Cell Death in Response to ER Stress
Source: Front Plant Sci. 2018 Mar 16;9:348. doi: 10.3389/fpls.2018.00348 (PMC5864889; doi:10.3389/fpls.2018.00348)
Supplement: Supplementary file 1 [file Data_Sheet_1.PDF]

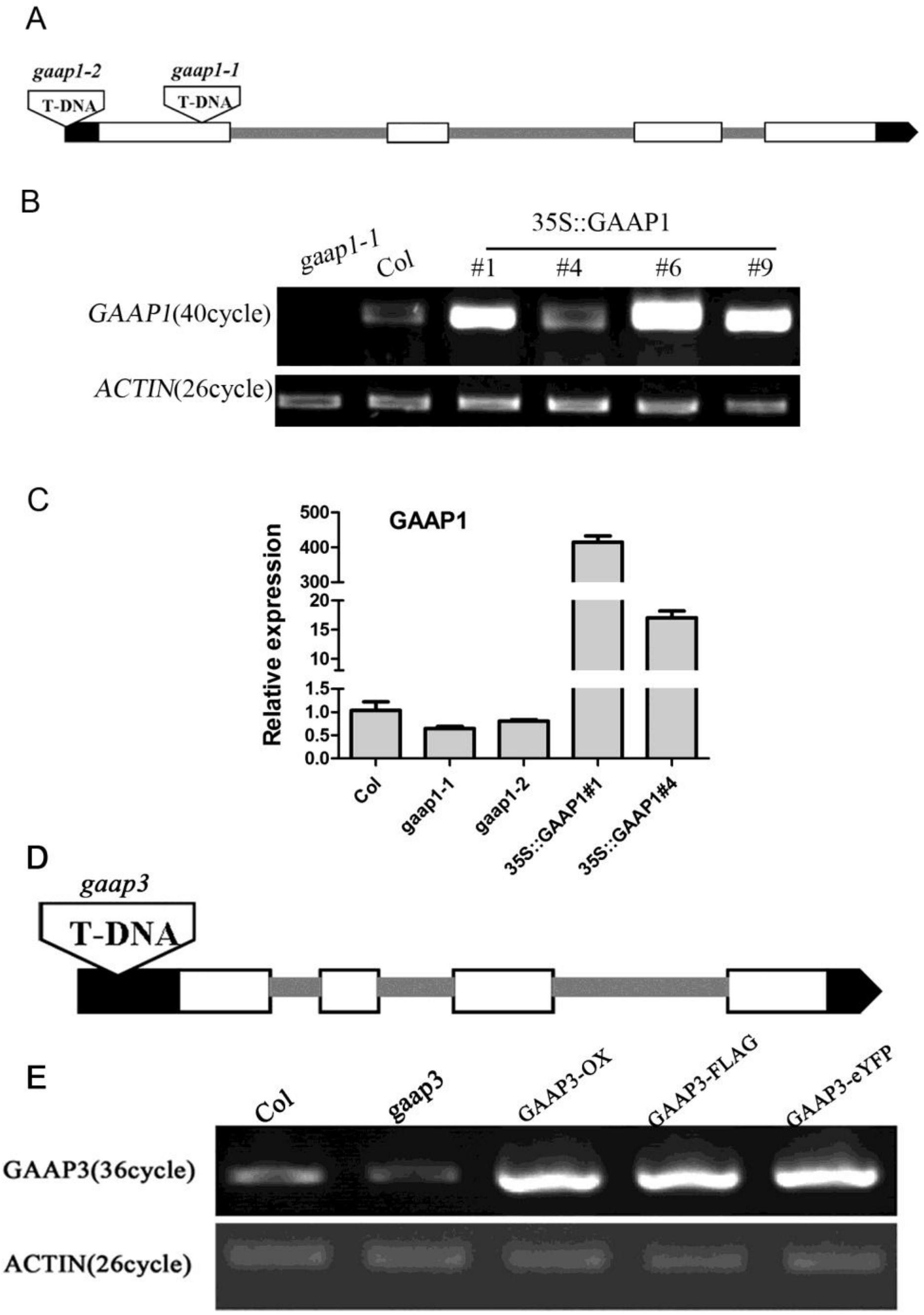

Supplementary Figure S1 GAAP1 and GAAP3 transcripts level in mutant and transgenic lines.

(A) Schematic representation of T-DNA insertion sites in *gaap1-1* and *gaap1-2*. White and black boxes indicate coding sequences and untranslated regions, respectively. T-DNA insertion site of *gaap1-2* is located in the promoter of the gene.

(B) Expression levels of *GAAP1* in the WT, three independent transgenic lines overexpressing *GAAP1* and *gaap1-1* were determined by RT-PCR using total RNA isolated from 2-week-old seedlings.

(C) Expression levels of *GAAP1* in the WT, 2 independent transgenic lines overexpressing *GAAP1*, *gaap1-1* and *gaap1-2* were determined by qRT-PCR using total RNA isolated from 12-day-old

seedlings.

(D) Schematic representation of T-DNA insertion sites in *gaap3*. White and black boxes indicate coding sequences and untranslated regions, respectively.

(E) Expression levels of *GAAP3* in the Col, three independent transgenic lines overexpressing *GAAP3* and *gaap3* were determined by RT-PCR using total RNA isolated from 2-week-old seedlings.

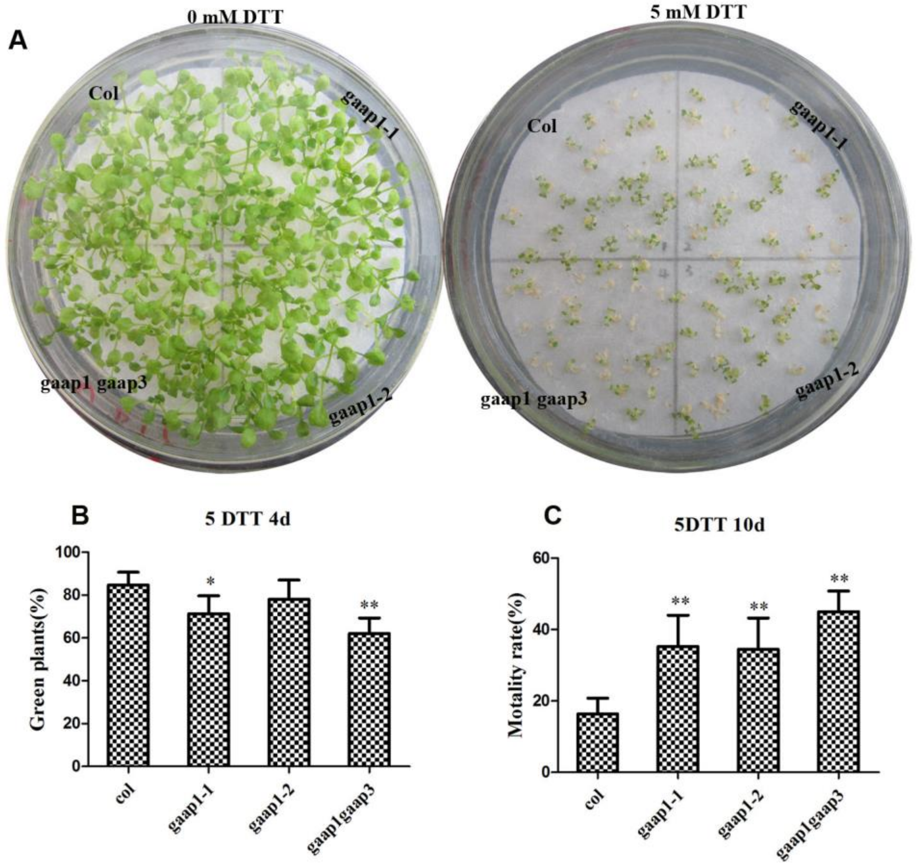

Supplementary Figure S2 Response of Col, *gaap1-1*, *gaap1-2* and *gaap1gaap3* to different concentrations of DTT. Four- day-old seedlings were transferred to medium supplied with or without 5 mmol L^{-1} DTT. Photos were taken for an additional 10 days **(A)**. The percentage of green plants after 4 days treatments **(B)** and the mortality after 10 days treatments with DTT **(C)** were calculated respectively. Error bars represent the standard error. Significant differences compared with Col plants were indicated by asterisk（χ test, *p<0.05, ** p<0.01, n = 6）.

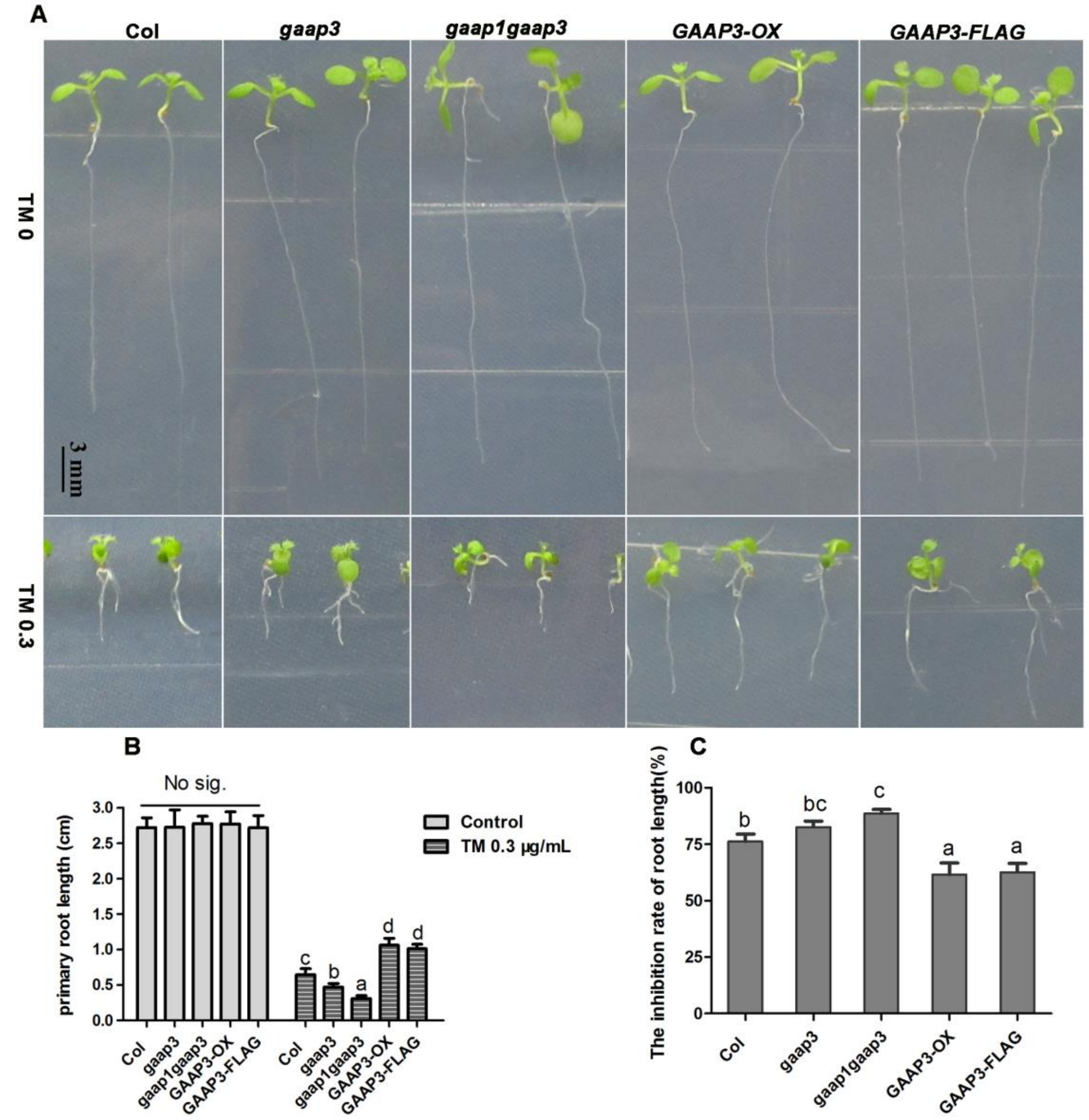

Supplementary Figure S3 ***GAAP3*** **single mutation or GAAP3 and GAAP1 double mutations enhanced the inhibition root growth, while over-expressing GAAP3 reduced the inhibition root growth upon ER stress.**

(A) The plants cultured on the medium for 6 days in the absence and presence of TM. The seeds germinated on the normal medium for 2days and then transferred to the medium containing different concentration of TM to . **(B)** The primary root length of plants growing on medium containing different concentration of TM for 8 days. **(C)** The inhibition rate of root length by TM showed in **(B)**. Error bars represent the standard deviation of 4 independent experiments. Different lowercase letters indicate significant differences between different plants (Tukey’s range test, $P < 0.05$, $n > 100$).

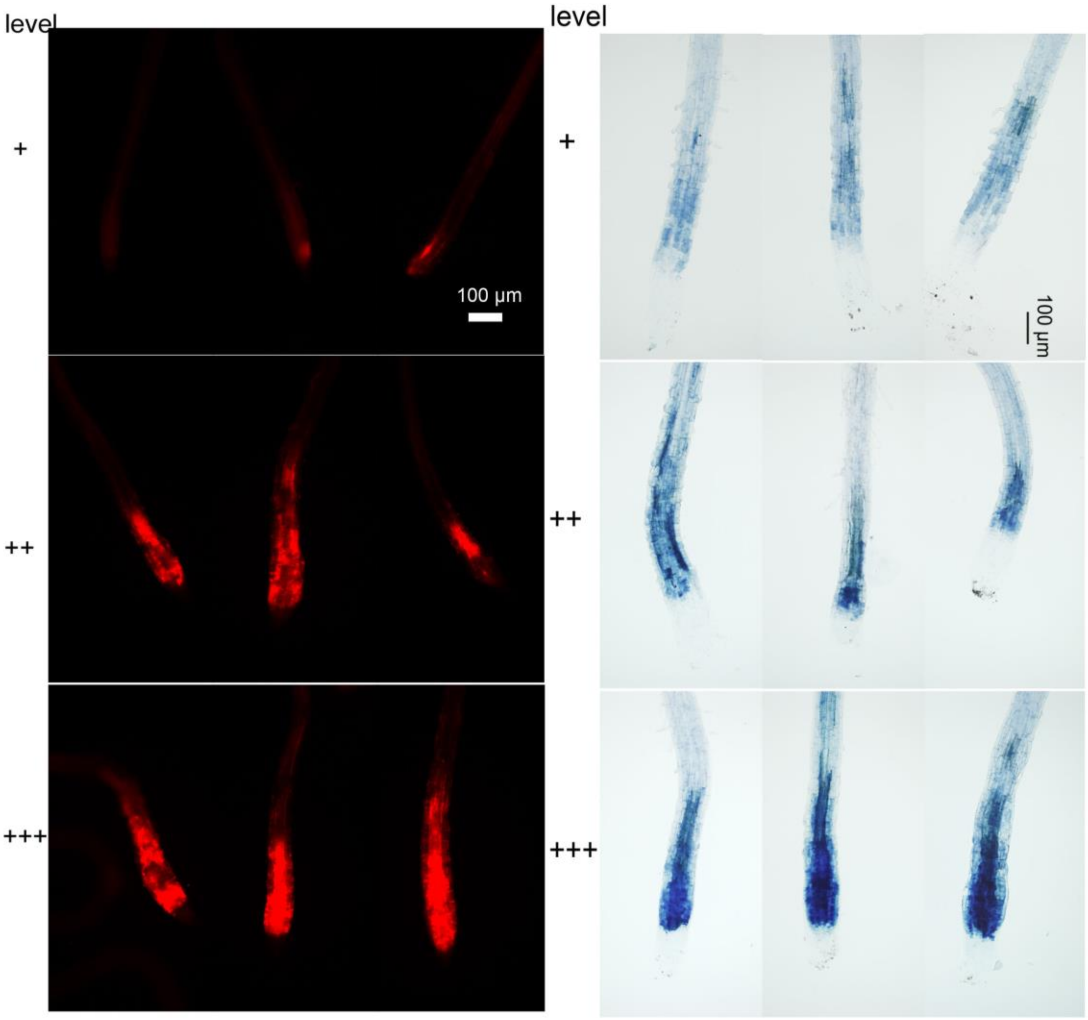

Supplementary Figure S4 The staining level of root cells stained by PI and trypan-blue upon TM treatment. Three-day-old seedlings which were vertically cultured were transferred to new culture plate containing different concentrations of TM (0, 0.15 and 0.30 μg mL^{-1}) and root cells were stained by PI and trypan blue after 48 h. The staining intensity were classified 3 level, faint, medium and strong depicted as "+, ++ and +++" respectively. Bar = 100 μm.

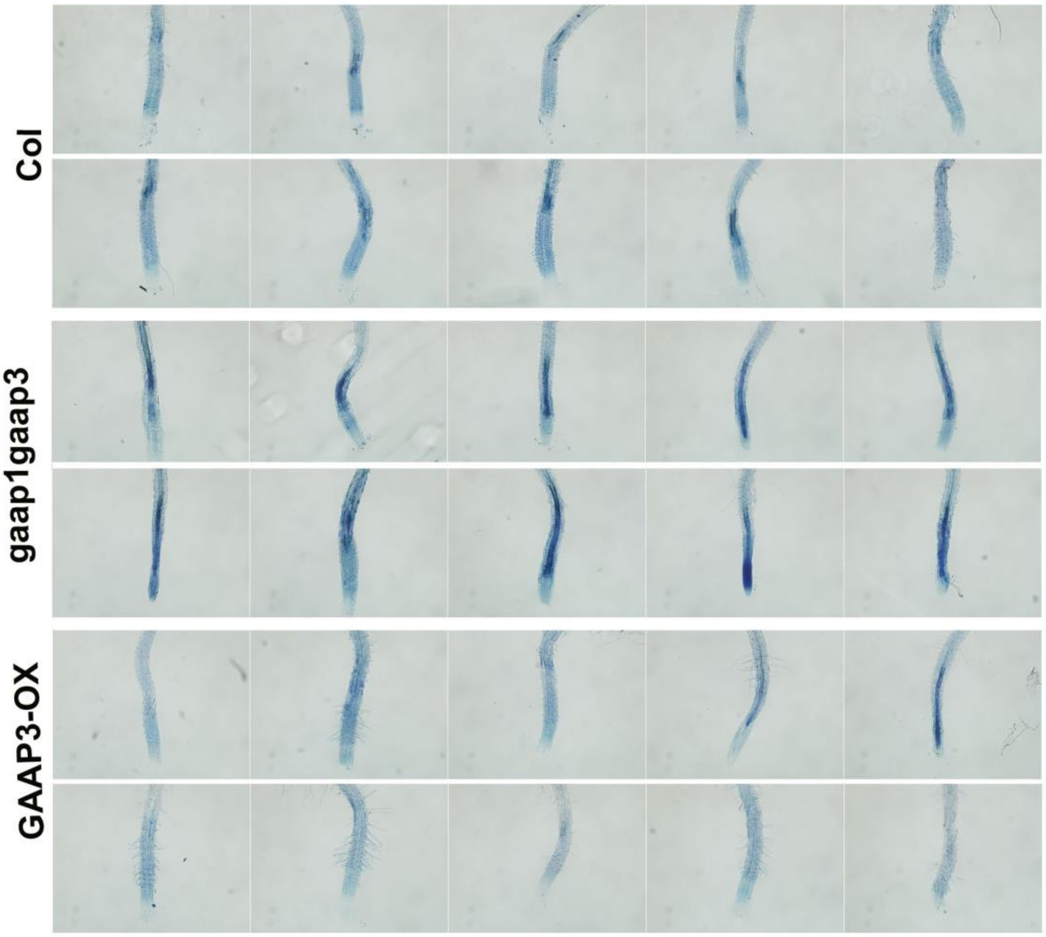

Supplementary Figure S5 The staining intensity of root cells of various plants treated with TM. Three-day-old seedlings which were vertically cultured were transferred to liquid medium containing 0.3 μg mL^{-1} TM for 48 h. And root cells of Col, *gaap1gaap3*and 35S::GAAP3 were stained by trypan-blue.

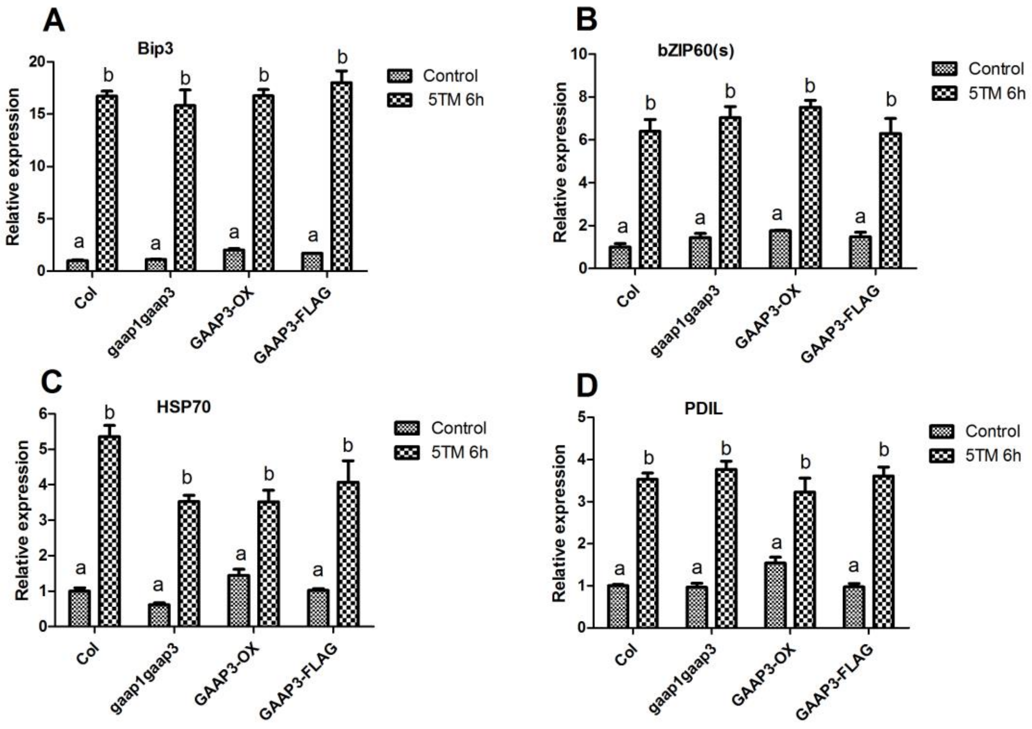

Supplementary Figure S6 Mutation of *GAAP1* and *GAAP3* or overexpressing *GAAP3* had no effect on the induction of UPR genes upon acute ER stress.

(A-D) The induction of UPR marker genes in Col, *gaap1gaap3*, GAAP3-overexpressing plants upon acute ER stress assayed by qRT. Total RNA was isolated from 7 days–old Arabidopsis seedlings that had been infiltrated with 1/2 MS liquid salt containing 5.0 μg/mL Tunicamycin for 6 h. Seedlings infiltrated with 1/2 MS liquid salt containing 0.1% DMSO were used as control. The transcript levels of selected ER marker genes, *Bip3, bZIP60(s), HSP70* and *PDIL1*, were quantified by qRT–PCR. The relative gene expression was the transcript level of each gene in different genotype plants normalized to the level in the wild-type control, both of which were normalized to expression of ACTIN8. Data are from 3 to 4 biological replicates (±SD). Statistically significance was analyzed according to Tukey's range (honestly significant difference) test and two-way analysis of variance ($p < 0.05$).

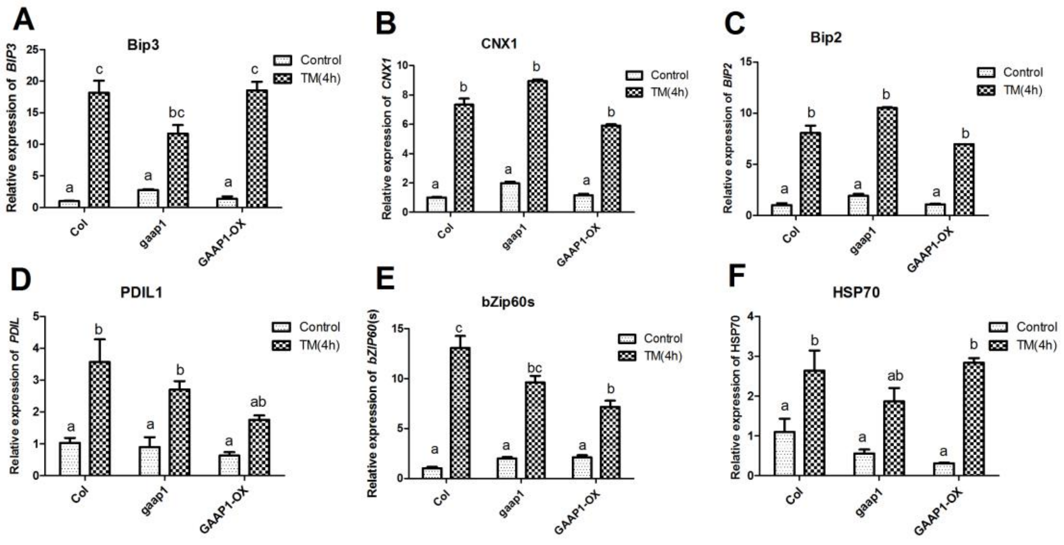

Supplementary Figure S7 *GAAP1* mutation or overexpressing had little effect on the induction of UPR genes upon acute ER stress. Total RNA was isolated from 7 days–old seedlings of Col, *gaap1-1* and 35S::GAAP1-OX#4 were treated with 5.0 µg/mL Tunicamycin or 0.1% DMSO (control) for 4 h. The transcript levels of selected ER marker genes were quantified by qRT–PCR. Gene expression was normalized to ACTIN8 and the value of each control of WT (Col) was set at 1. Data are from 3 to 4 biological replicates (±SD). Statistically significance was analyzed according to Tukey's range (honestly significant difference) test and two-way analysis of variance ($p < 0.05$).

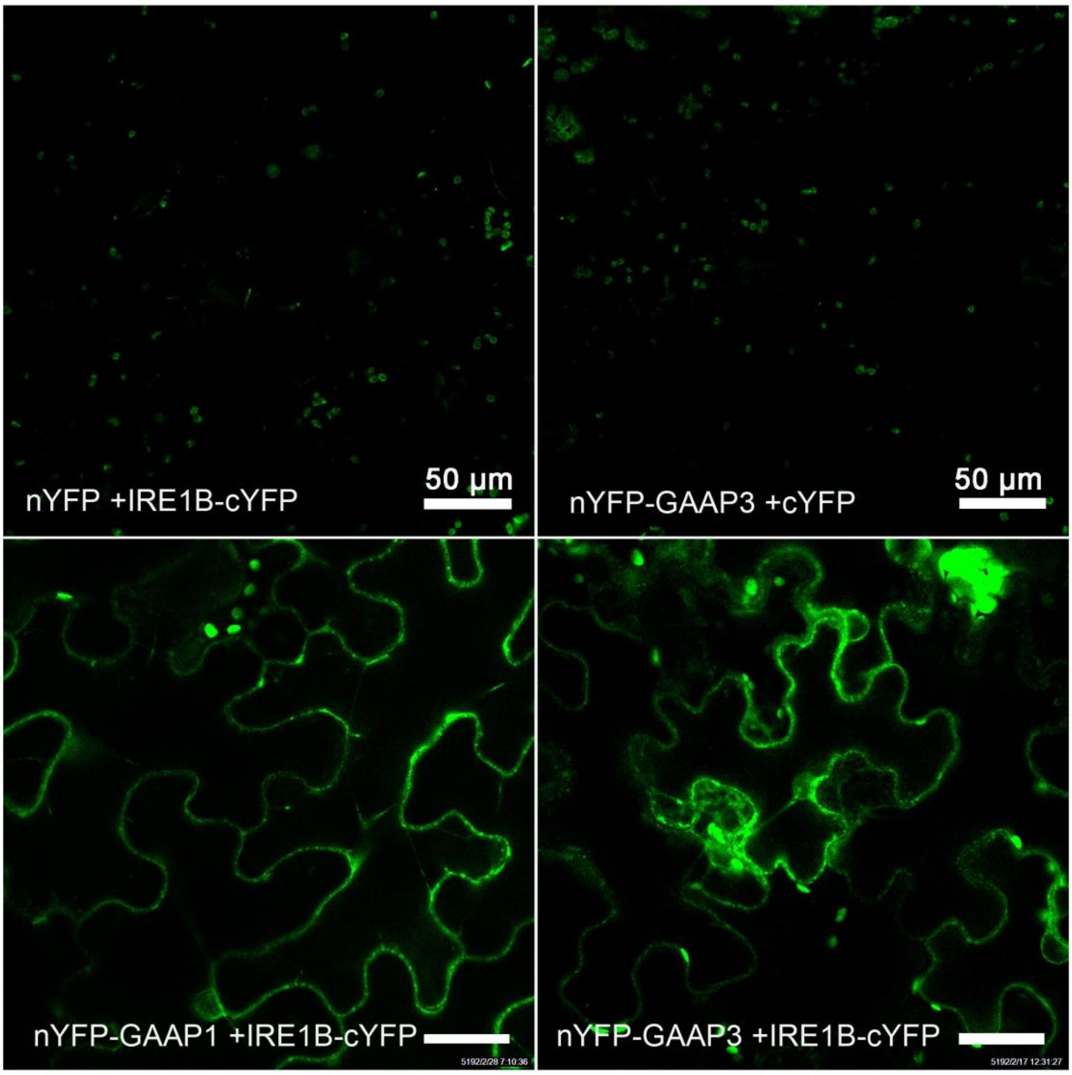

Supplementary Figure S8 GAAP1/GAAP3 interacted with IRE1B, as showed by BiFC assay. YFP fluorescence was observed when nYFP-GAAP1/GAAP3 and IRE1B-KR-cYFP were co-expressed in the tobacco leaf cell, while only the background signal of chlorophyll were observed when IRE1B-KR-cYFP and nYFP were co-expressed, or nYFP-GAAP1/GAAP3 and cYFP were co-expressed (here only the picture of nYFP-GAAP3 with cYFP showed).

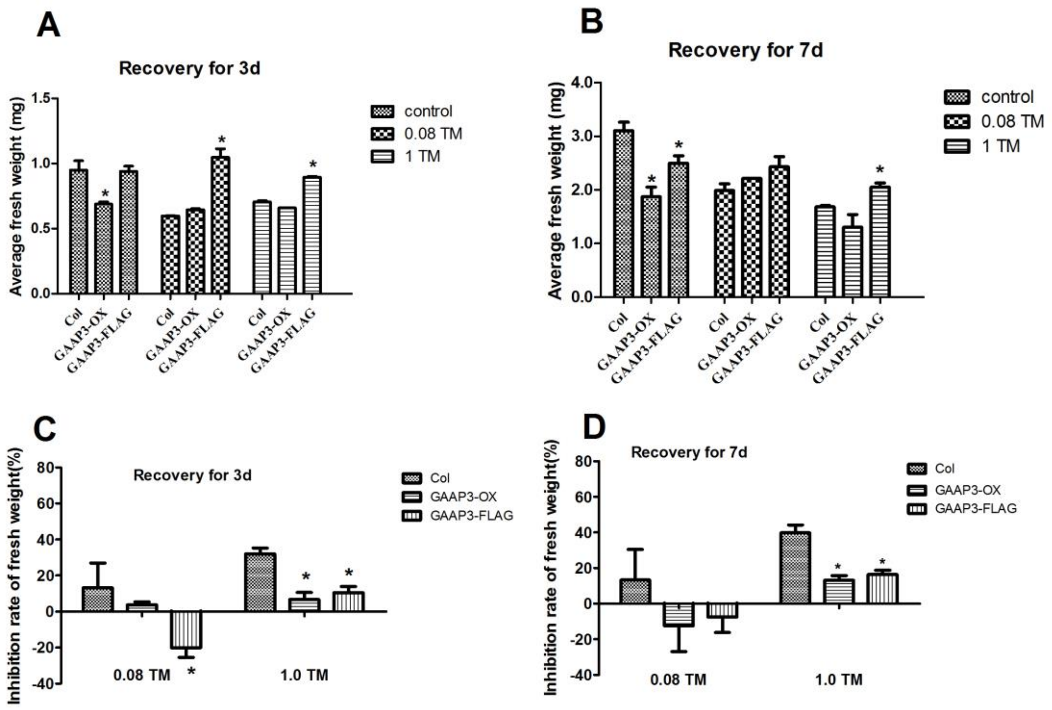

Supplementary Figure S9 GAAP3 promoted the growth recovery after the mild ER stress was relieved.

(A-D) The 4 days–old Arabidopsis seedlings that had been infiltrated with 1/2 MS liquid salt containing 0, 0.08 and 1.0 μg mL^{-1} TM for 6 h respectively were transferred to the 1/2 MS solid medium. And the fresh weight of seedlings (A, B) and the inhibition rate (C, D) was determined after recovery for 3 d and 7 d. Data are from three biological replicates (±SE) and at least 30 samples for each plant line each treatment. Asterisks refer to significant differences from Col at the same time point and same condition (*t*-test; $p < 0.05$).

Supplementary Table S1. Primers used.

gene/mutant/ constructs name	T-DNA lines/AGI number	Purpose	Primer name	Primer sequences (5' to 3')
gaap1-1	Salk_046652	Genotyping	LP	TCCTTTCCATTTGCATATATTTTG
			RP	ACAATGCAGTTTATCGGAT

				GC
gaap1-2	CS814417	Genotyping	LP1	GCAGAGTTGCAGAAACAGGAG
			RP1	TTGGTGGCTGATAAGGAACAG
gaap3	SALK_001992	Genotyping	*LP*	TTGATGTGTCCAAAGGGAAA
			RP	CTCGACCAAACCCTAACCTTC
35S::GAAP1	At4g14730	Protein expression in plant	F2	gaattcactagtctcgagATGGCGAAATCTGACATTGAAAC
			R2	gaattcggatccggcgcgccTCAGTTGCTTATAATGCCGAGG
35S::GAAP1-YFP/CFP	At4g14730	Protein expression in plant	F11	aagcttATGGCGAAATCTGACATTGAAAC
			R8	ggatccGTTGCTTATAATGCCGAGG
PGAAP1::GUS	At4g14730	GUS assay	PF1	cgactctagaggatccAAATGAACAGAAAATGAGAAGTG
			PR1	gaccacccggggatccTTGTTGTTGAAATCGCAAA
35S::GAAP3/35S::eYFP-GAAP3	AT4G02690	Protein expression in plant	F1	gaattcactagtgtcgacATGTATCAGTGGAACTTACCGTACC
			R1	gaattcggatccggcgcgccTCATCTCTGTAAGGCTCTCAACA
35S::GAAP3	AT4G026	Protein	F8	ctcgagggatccATGTATCAGTGG

-YFP/CFP	90	expression in plant		AACTTACCGTACC
			R10	ctgcagTCTCTGTAAGGCTCTCAACA (PstI)
PGAAP3::GUS	AT4G02690	GUS assay	PF1	ggatccaagcttgtcgacAATTATCTGAGAGCTCTTTTGTTTCA(BamHI-Hind III-SalI)
			PR1	ggatccGAAAATTCGAATCCTTTCGATTAC(BamHI)
35S::CFP-HDEL		cellular localization	FP1	5'-TCCcccgggATGGTGAGCAAGGGCGAGGAG-3'
			RP1	5'-GgaattcTTACAGCTCGTCATGAGATCTCTTCTT-3'
35S::FLAG-GAAP3	AT4G02690	Co-IP	F8	CTCGAGGGATCCATGTATCAGTGGAACTTACCGTACC
			R8	GAATTCGTCGACTCATCTCTGTAAGGCTCTCAACA
35S::IRE1a-KR-TAP	AT2G17520	Co-IP	*F9*	agaactagtggatccATGCCAGTAAAGTTATTGGCCCCTTT
			R2	ctttcccatggatccGATGATGTCGCATTTGAAG
IRE1A-KR-cYFP	**AT2G17520**	BiFC	**IRE1a** F16	acaggtacccggggatcc ATG **cCAGTAAAGTTATTGGCCCCTTT**
			**IRE1a** R16	gtcgactctagaggatcc GATGATGTCGCATTTGAAG
nYFP-GAAP1	At4g14730	BiFC	*At4g14730F12*	CGggatccATGGCGAAATCTGACATTGAAAC
			At4g14730R12	**ACGtctagaTCAGTTGCTTATAATGCCGAGG (XbaI)**
nYFP-GAAP	AT4G02690	BiFC	**At4g02690F12**	CGggatcc ATGTATCAGTGGAACTTACCG

3				TACC
			**At4g026 90R13**	gtcgac**tctaga TCA**TCTCTGTAAGGCTCTCAA CA
IRE1B-KR-c YFP	AT5G24360	BiFC	IRE1bF1 6	acaggtacccggggatcc ATG ctaattTTCGGCTTTTGTGTTA CTATGC
			IRE1bR1 6:	gtcgactctagaggatcc GAATACAGTGGTCTTAGAG TA
BiP3	At1g09080	qRT-PCR	BiP3 F1	CGAAACGTCTGATTGGAAGA A
			BiP3 R1	GGCTTCCCATCTTTGTTCAC
ACT8	AT1G492 40	qRT-PCR	Act8 F1	TCAGCACTTTCCAGCAGATG
		qRT-PCR	Act8 R1	ATGCCTGGACCTGCTTCAT
bZIP60	AT1G429 90	qRT-PCR	bZIP60 F4	GAAGGAGACGATGATGCTGT GGCT
		qRT-PCR	bZIP60U B1	GCAGGGATTCCAACAAGAGC ACAG
		qRT-PCR	bZIP60S B2	AGCAGGGAACCCAACAGCAG ACT
PDIL1	At1g2175 0	qRT-PCR	qPDIL1F	CTCGTGAAGCTGAGGGTATTG
		qRT-PCR	qPDIL1R	TGTGCGAAATCTAACTCAGAG
SHD	AT4G241 90	qRT-PCR	qSHD F	GAAGGAAGCATTCAAGGAGC TA
		qRT-PCR	qSHD R	TCTTTGATGATAGGGTGTCGT G
HSP70	AT4G166 60	qRT-PCR	qHSP70 F	GGACTTGGAGGACAGAGTAT GG
		qRT-PCR	qHSP70 R	GGGTAATTGTGCTCCTGAAGT C
NAC103	AT5G640 60	qRT-PCR	qNAC10 3F	AAGTCGTAGAGCTACAGGAT
		qRT-PCR	qNAC10	TAGGAAACGTTCTGGCTAAA

			3R	G
CNX1	At5g61790	qRT-PCR	CNX1 qFW	TTCTTCTCGCTCTTCCTCAAGC
		qRT-PCR	CNX1 qRV	GCGGTTTCTTCCTTCTTCTCC
